# Rational Design of Mn-APTES/1-Methylimidazole Nanozymes: Enhanced Laccase-like Activity at Near-Neutral pH for Environmental Remediation

**DOI:** 10.3390/ijms27062583

**Published:** 2026-03-11

**Authors:** Almendra Fernández, Ana Obreque, Olga Rubilar, Edward Hermosilla

**Affiliations:** 1 Chemical Engineering Department, Universidad de La Frontera, Temuco 4811230, Chile; 2 Biotechnological Research Center Applied to the Environment (CIBAMA-BIOREN), Universidad de La Frontera, Temuco 4811230, Chile; 3 Instituto de Ciencias Aplicadas, Universidad Autónoma de Chile, Temuco 4810101, Chile

**Keywords:** nanozymes, laccase-like activity, synthesis optimization, environmental remediation, manganese, oxytetracycline

## Abstract

Natural laccases are a widely reported option for pollutant degradation; however, their widespread application is severely restricted by high production costs, limited storage stability, and rapid inactivation at the neutral pH typical of wastewater treatment plants. To overcome these limitations, we rationally designed manganese-based nanozymes (Mn-APTES/1MeIm) that mimic natural metal–histidine coordination within a protective siloxane network. Optimization via Response Surface Methodology produced two variants, Mn-APTES/1MeIm-6 and Mn-APTES/1MeIm-7, revealing distinct synthesis mechanisms: catalytic activity at pH 6 is driven by synthesis temperature, whereas activity at pH 7 is controlled by the APTES:1MeIm molar ratio. TEM and XRD analysis confirmed a delaminated aminoclay architecture composed of electron-transparent nanosheets, while FTIR verified Mn–N coordination through characteristic blue shifts. The optimized nanozymes retained robust activity, exhibiting maximum reaction velocities of 4.331 µM min^−1^ (Mn-APTES/1MeIm-6) and 1.71 µM min^−1^ (Mn-APTES/1MeIm-7), whereas *Trametes versicolor* laccase was practically inactive. Practically, Mn-APTES/1MeIm-6 achieved 75% degradation of oxytetracycline in 120 min without detectable manganese leaching, significantly outperforming the natural enzyme (<13%). These findings present a robust, pH-stable alternative for sustainable environmental remediation.

## 1. Introduction

The prevalent problem of environmental contamination, particularly in wastewater, demands the development of advanced, sustainable, and efficient remediation solutions. While conventional treatment technologies often face limitations in terms of cost, energy consumption, and secondary pollution [1,2], enzymatic methods have gained attention because they offer a green alternative in bioremediation of wastewater. In particular, laccase enzymes have shown promise as they can degrade a wide range of phenolic and non-phenolic compounds, including tetracyclines [3]. Studies have shown that laccases can effectively degrade tetracycline, even in complex matrices like seawater, by converting it into less toxic byproducts [4,5]. Nevertheless, their inherent limitations, including low stability, high cost, and sensitivity to environmental conditions, require the exploration of robust alternatives [6].

To address the drawbacks of natural laccase, there is a growing interest in artificially engineered nanozymes. These nanomaterials are designed to mimic the catalytic functions of natural enzymes, including laccase-like activity. Laccase-like nanozymes present a notable advantage due to their ability to catalyze oxidation reactions without the need for external oxidants such as hydrogen peroxide (H_2_O_2_), which is essential for the activity of peroxidase-like nanozymes. This intrinsic oxidative capability makes laccase-like nanozymes particularly attractive for environmentally friendly and sustainable pollutant degradation strategies. Among these, metal- and metal oxide-based nanozymes are the most commonly reported due to their inherent redox properties [7,8,9]. Currently, these nanozymes have been extensively explored for their sensing applications across food safety, clinical diagnostics, and environmental monitoring, enabling the detection of various substances such as phenolic compounds, pesticides, neurotransmitters, antibiotics, enzymes, and heavy metals [10]. In general, nanozymes have been less commonly employed for the degradation of organic pollutants compared to sensing. Recent findings have demonstrated that laccase-like nanozymes have considerable potential in degrading aromatic pollutants. For instance, MnFe_2_O_4_@Mn_3_O_4_|CuO laccase-like nanozymes achieved 100% degradation of methyl orange within 15 min at pH 5 [11]. Cu–Mn aminoclay nanozymes exhibited 95.1% degradation of bisphenol A after 24 h at pH 3 [12]. Other transition metal-based nanozymes have also demonstrated degradation capabilities associated with laccase-like activity. MnO_2_ nanozymes achieved 97.3% degradation of 17β-estradiol within 4 h [13]. PtCo@DMSN nanozymes exhibited 81.83% degradation of phthalic acid esters (commonly used as plasticizers) after 72 h [14].

Despite the demonstrated potential of laccase-like nanozymes, these materials often face significant limitations in terms of stability and practical applicability, particularly for real-world environmental remediation. A major challenge lies in their restricted operational pH range. Specifically, laccase-like nanozymes based on metals or metal oxides typically exhibit optimal activity under highly acidic conditions (pH 3–5). Under such conditions, metal leaching can occur, leading to the deactivation of the nanozyme and causing secondary contamination risks, which is particularly problematic in wastewater treatment applications [11]. Moreover, natural water sources and most wastewater streams typically have neutral to slightly alkaline pH levels (6–8). During treatment in WWTPs the pH is maintained around 7–7.4, which further limits the effectiveness of conventional laccase-like nanozymes under real-world conditions [15]. Therefore, developing stable, efficient nanozymes that operate under broader and more environmentally relevant pH conditions is essential for practical applications in environmental remediation.

Although natural laccases and the majority of laccase-mimicking nanozymes rely on copper-based active sites, this design strategically employs manganese to address both environmental and catalytic limitations [16,17,18,19]. While copper is the natural cofactor, its accumulation presents potential toxicity risks in aquatic ecosystems, making manganese (Mn) a ‘greener’ and more biocompatible alternative for sustainable remediation [20]. Furthermore, Mn has a higher standard redox potential of the Mn^2+^/Mn^3+^ couple (1.50 V) compared to the traditional T1 copper center (~0.80 V) [21]. This significantly higher thermodynamic driving force is critical for the remediation of recalcitrant pollutants, as it enables the oxidation of stable aromatic compounds that typically resist lower-potential catalysts, thereby effectively expanding the catalytic scope beyond standard phenolic substrates. However, realizing this high oxidative potential at neutral pH requires overcoming the inherent tendency of manganese to precipitate as inactive hydroxides. Consequently, the design of a mixed complex incorporating manganese, 1-methylimidazole (1MeIm), and (3-aminopropyl)triethoxysilane (APTES) is driven by a dual-stabilization strategy intended to preserve the active Mn^2+^/Mn^3+^ cycle under near-neutral pH conditions. First, 1MeIm serves as a structural surrogate for the histidine residues found in natural active sites [22]. By coordinating directly with the Mn, it electronically modulates the metal center while its short methyl chain minimizes steric hindrance, thereby optimizing electron transfer rates [23]. Second, the APTES siloxane network creates a buffered interfacial microenvironment that shields the active site from bulk pH fluctuations [21]. This confinement prevents the formation of inactive manganese hydroxides (Mn(OH)_2_) and maintaining the metal in a dispersed and catalytically competent state even in neutral aqueous media [12].

Herein, we report the rational design and optimization of this Mn-APTES/1MeIm nanozyme via Response Surface Methodology (RSM). The optimized catalyst was subsequently compared with a commercial laccase from *Trametes versicolor* and validated through the degradation of oxytetracycline.

## 2. Results and Discussion

### 2.1. Optimization of Mn-APTES/1MeIm Nanozyme Synthesis

In this study, manganese-based nanozymes were prepared via a modified coprecipitation method. The design focused on mimicking the natural metal–histidine coordination of laccases by using 1-methylimidazole (1MeIm) as a ligand, stabilized within an aminoclay-like network formed by APTES. The optimization, structural properties, and catalytic capabilities of these materials are discussed in the following sections.

The synthesis of Mn-APTES/1MeIm nanozymes was systematically evaluated via a response surface methodology (RSM) utilizing a central composite design (CCD) (Table 1) to maximize the laccase-like activity at near-neutral pH (6, 7 and 8). While maintaining the Mn precursor fixed at 1.5 mmol, the experimental design probed the structural impact of varying the concentrations of the organic ligands, APTES (3–9 mmol) and 1MeIm (3–9 mmol), as well as the synthesis temperature (30–50 °C). Preliminary screening restricted the optimization to pH 6 and pH 7, as the synthesized nanozymes exhibited negligible catalytic activity at pH 8 (0.025–0.225 U mg^−1^) and failed to fit quadratic models. The optimization of synthesis conditions for a nanozyme with maximum laccase-like activity at pH 7 revealed a synthesis process governed primarily by chemical stoichiometry rather than thermal kinetics. Regression analysis yielded a statistically robust predictive model that successfully captured the variance in the catalytic response (Table 2). The predictive quadratic polynomial models for laccase-like activity in terms of coded factors are expressed by Equation (1):

Activity (pH 7) = 1.175 + 0.085*X*_1_ − 0.250*X*_2_ − 0.048*X*_3_ + 0.275*X*_1_*X*_2_ + 0.038*X*_1_*X*_3_ − 0.031*X*_2_*X*_3_ − 0.494*X*_1_^2^ + 0.156*X*_2_^2^ − 0.182*X*_3_^2^ (1)

Within this neutral environment, the concentration of the imidazole ligand proved pivotal, exhibiting a statistically significant negative linear correlation (*p* < 0.05). Although the imidazole moiety is essential for mimicking the active site of laccase via N-Mn or N-Cu coordination, this negative trend indicates that excessive organic loading likely induces a “structural shielding” effect [20,24,25]. High ligand concentrations appear to reduce the density of exposed active sites per unit area, effectively saturating adsorption points with ligand molecules rather than substrate. Conversely, the silane precursor demonstrated a dominant quadratic influence rather than a linear one, indicating a convex activity profile where stoichiometric deviations significantly diminish performance. Furthermore, a pronounced interaction between APTES and 1MeIm underscored the necessity of a precise ligand-to-linker ratio to effectively modulate the metal coordination. Notably, thermal variations within the 30–50 °C range did not significantly alter the catalytic outcome at this pH (*p* > 0.05). In contrast, the synthesis optimization for maximum laccase-like activity at pH 6 demonstrated a distinct shift from stoichiometric control to pronounced thermal sensitivity. The resulting model for pH 6 identified synthesis temperature as the sole critical determinant (Table 2). Similarly, the predictive model for laccase-like activity at pH 6 is expressed by Equation (2):

Activity (pH 6) = 0.457 + 0.093*X*_1_ − 0.233*X*_2_ − 0.643*X*_3_ − 0.047*X*_1_*X*_2_
*−* 0.09*X*_1_*X*_3_ + 0.128*X*_2_*X*_3_ − 0.180*X*_1_^2^ + 0.195*X*_2_^2^ + 0.870*X*_3_^2^ (2)

Both linear (*p* = 0.003) and quadratic (*p* = 0.017) thermal terms exhibited statistical significance, indicating that deviations from the lower temperature range rapidly reduce the potential catalytic performance. This effect aligns with established literature regarding the stability of laminar nanozymes, where the assembly relies critically on the formation energy of metal-oxygen-silane bonds [26]. Deviations from the identified lower temperature optimum (30 °C) likely precipitate thermodynamic instability, leading to the deconstruction or aggregation of the structures [27]. Furthermore, while moderate temperatures favor the formation of high-surface-area nanoparticles, excessive heat induces a conformational rigidity that restricts the catalytic dynamics necessary for effective substrate binding.

In contrast to the results at pH 7, neither the concentration of APTES (*p* = 0.544) nor 1MeIm (*p* = 0.153) showed a significant direct impact on the response, and no interaction effects were detected. The distinct governance mechanisms observed for the two optimized variants, control by synthesis temperature for the pH 6-active nanozyme versus control by the APTES:1MeIm molar ratio for the pH 7-active nanozyme, reflect the different stability requirements of the Mn center in the final operational environment. For the nanozyme optimized for slight acidic conditions (pH 6), the Mn center is inherently soluble and chemically stable; therefore, the synthesis appears to be governed by morphological kinetics. The lower synthesis temperature (30 °C) likely favors the formation of high-surface-area, exfoliated nanosheets (kinetic product), whereas higher synthesis temperatures induce aggregation or excessive condensation of the siloxane network, reducing the accessible active sites required for operation at pH 6. Conversely, for the nanozyme optimized for neutral conditions (pH 7), the primary challenge is preventing the thermodynamic precipitation of manganese hydroxides. Consequently, the synthesis becomes stoichiometrically controlled, as the precise ratio of 1MeIm to Mn is critical to form a robust coordinate complex that can chemically shield the metal center. In this regime, the ‘chemical protection’ provided by the optimal ligand concentration outweighs the morphological effects of temperature, ensuring the catalyst remains active even in a neutral environment where the bare metal would typically deactivate.

**Figure 1:**
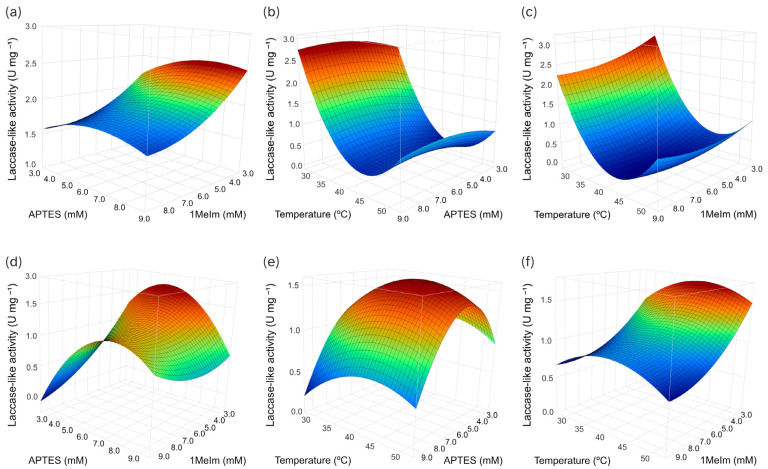
Response surface optimization of Mn-APTES/1MeIm nanozyme synthesis parameters. Three-dimensional plots illustrate the interactive effects of APTES and 1MeIm concentrations, and synthesis temperature on laccase-like specific activity (U mg^−1^) at (**a**–**c**) pH 6 and (**d**–**f**) pH 7.

The interactive effects of precursor concentration and synthesis temperature on catalytic performance are visually corroborated by the surface topologies presented in Figure 1. Specifically at pH 7 (Figure 1d–f), the response surface is characterized by a convex profile driven by the stoichiometry of the organic ligands. This curvature confirms that while the silane network is essential for matrix stability, increasing the 1MeIm concentration beyond the optimum leads to a sharp decline in activity, attributable to steric hindrance obstructing the manganese coordination sites. Conversely, the response surfaces for pH 6 (Figure 1a–c) reveal a landscape dominated exclusively by thermal parameters. The steep gradients observed along the temperature axis, independent of ligand variation, indicate that elevated synthesis temperatures disrupt the formation of the active metallosurfactant structure.

To validate the statistical reliability of the response surface models and confirm the theoretical optima, an analysis of variance (ANOVA) and experimental verification were conducted. The statistical summary presented in Table 2 corroborates the robustness of the regression models for both pH conditions. The model for neutral pH (pH 7) demonstrated a high coefficient of determination (R^2^ = 0.864) and a significant F-ratio (*p* = 0.023), indicating that the quadratic polynomial successfully captures the complex stoichiometric interactions governing the synthesis. Similarly, the model for pH 6, while slightly less predictive (R^2^ = 0.848), remained statistically significant (*p* = 0.033) and sufficient to navigate the design space. The proximity of the adjusted R^2^ values to the experimental R^2^ in both cases suggests that the models are free from significant insignificant terms and provide a reliable approximation of the true response surface. Based on these predictive landscapes, the optimal synthesis parameters were experimentally validated to assess the accuracy of the computational projections (Table 3). For the slight acidic variant (Mn-APTES/1MeIm-6), the model predicted a maximum laccase-like specific activity of 2.55 U mg^−1^ using 7.24 mM APTES and 3 mM 1MeIm at 30 °C, corresponding to a Mn:APTES:1MeIm molar ratio of 1:4.83:2. The experimental validation yielded a specific activity of 2.23 U mg^−1^, achieving a high degree of concordance with the theoretical value. Similarly, for the neutral variant (Mn-APTES/1MeIm-7), the optimization model projected a maximum activity of 1.61 U mg^−1^ utilizing 5.45 mM APTES and 3 mM 1MeIm at 42 °C, representing a molar ratio of 1:3.63:2. The experimental verification confirmed this prediction with a specific activity of 1.45 U mg^−1^, validating the model’s capacity to tailor the catalyst for neutral pH operation. The predictive capability of these models is statistically comparable to similar nanozyme and nanocatalysts optimization studies employing Box–Behnken designs [28,29,30].

### 2.2. Specific Activity of Natural Laccase and the Optimized Nanozymes Under Near-Neutral pH

**Figure 2:**
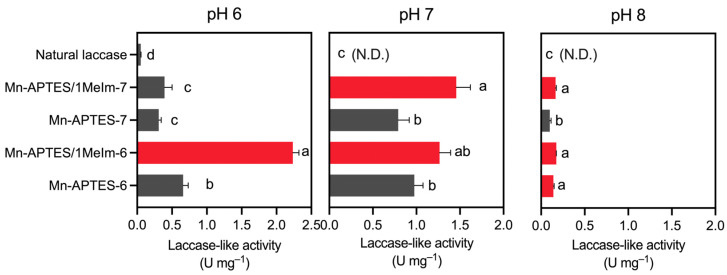
Comparative catalytic performance of optimized Mn-APTES/1MeIm nanozymes versus natural laccase at near-neutral pH. Error bars indicate the standard deviation (*n* = 3). Different letters indicate statistically significant differences (*p* < 0.05) according to Tukey’s HSD post hoc test.

To evaluate the potential of Mn-APTES/1MeIm nanozymes as a robust alternative to natural laccase and elucidate the specific influence of the imidazole ligand on catalytic performance, the specific laccase-like activity of the optimized nanozymes was systematically compared with their ligand-free counterparts (Mn-APTES) and a commercial fungal laccase across a defined pH gradient (pH 6–8) (Figure 2). Notably, in this study, laccase-like activity was quantified via the chromogenic oxidation of ABTS over a 1.5 min interval. This reaction time contrasts with periods typically reported for comparable nanozymes in the literature, which often require incubation times of 20, 30, or even 60 min despite utilizing similar or higher catalyst loadings [31,32,33]. The ability to achieve significant substrate oxidation within a 1.5 min timeframe highlights an exceptionally high initial reaction rate. This rapid turnover is a critical advantage for practical environmental remediation, suggesting the material can process high pollutant loads rapidly compared to standard nanozymes. The results reveal a critical difference in catalytic activity between the synthetic mimetics and the natural enzyme.

The results reveal a critical difference in catalytic activity between the synthetic mimetics and the natural enzyme. At pH 6, the optimized Mn-APTES/1MeIm-6 yielded the maximal catalytic response, significantly surpassing the 1MeIm-free Mn-APTES-6 control by 2.3-fold. This enhancement underscores the role of the 1MeIm in facilitating electron transfer under near-neutral pH conditions. In contrast, the commercial laccase exhibited only marginal activity (0.05 U mg^−1^), while the variants optimized for pH 7, Mn-APTES/1MeIm-7 (0.4 U mg^−1^) and its control Mn-APTES-7 (0.3 U mg^−1^) performed poorly, highlighting the critical importance of the specific synthesis conditions in defining an active structural conformation tailored for pH-dependent operation. Although it is well-established that natural laccases are highly efficient at their optimal acidic pH, their activity drastically and rapidly decreases as the pH approaches neutral values (pH 6.0–7.0). The results presented herein aim to demonstrate that the designed Mn-APTES/1MeIm nanozyme provides a highly functional and robust alternative specifically for applications requiring near-neutral conditions, such as the treatment of municipal wastewater, where the natural enzyme becomes practically inactive.

The functional superiority of the nanozymes becomes most pronounced at pH 7. Consistent with the known fragility of natural biocatalysts, the commercial laccase exhibited non-detectable activity (N.D.) at pH 7, confirming its rapid inactivation outside acidic environments. Conversely, the chemically modulated Mn-APTES/1MeIm-7 achieved its peak performance at this neutral condition, effectively extending the functional window of laccase-like catalysis into physiologically relevant environments where the natural enzyme is inert. Notably, the Mn-APTES/1MeIm-6 variant displayed remarkable functional plasticity, retaining high catalytic activity at pH 7 that was statistically comparable to the Mn-APTES/1MeIm-7. Across both functional pH regimes, the imidazole-functionalized nanozymes consistently outperformed the bare Mn-APTES nanozymes, validating that the organic ligand acts as an active electronic modulator rather than a mere structural spacer. Finally, the uniform suppression of activity observed at pH 8 for all synthesized variants confirms the thermodynamic limits of the assembly in alkaline media, validating the limitations established during the preliminary optimization screening.

### 2.3. Kinetics Parameters of the Optimized Mn-APTES/1MeIm-6 and Mn-APTES-7 Nanozymes

**Figure 3:**
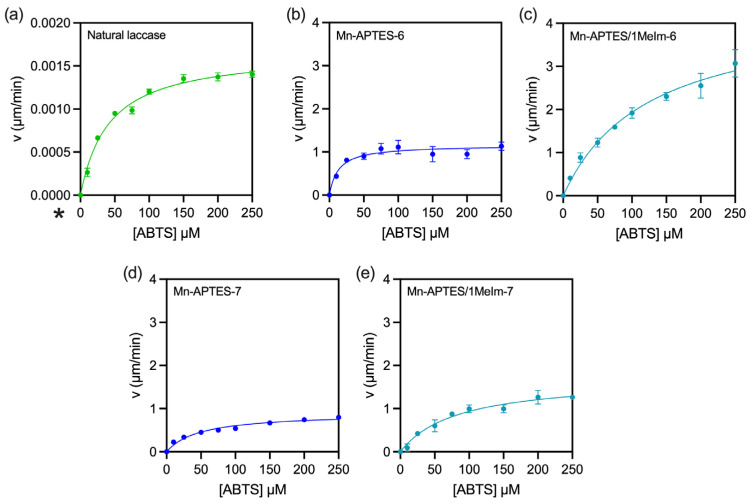
Steady-state kinetic profiles of the catalytic systems for ABTS oxidation. Michaelis–Menten plots displaying the initial reaction velocity (v) as a function of substrate concentration for (**a**) commercial laccase, (**b**) Mn-APTES-6, (**c**) Mn-APTES/1MeIm-6, (**d**) Mn-APTES-7, and (**e**) Mn-APTES/1MeIm-7. The solid lines represent the non-linear regression fits of the experimental data to the Michaelis–Menten equation. Error bars indicate the standard deviation (n = 3). * Indicates the distinct y-axis scale in (**a**), highlighting the significantly lower turnover rate of the natural enzyme compared to the synthesized nanozymes.

To elucidate the mechanistic influence of the ligand architecture on catalytic performance, steady-state kinetic assays were conducted for ABTS oxidation and fitted to the Michaelis–Menten model, as depicted in Figure 3. The quantitative parameters derived (Table 4) reveal a stark dichotomy between the synthetic mimetics and the commercial fungal laccase. At pH 6, the natural enzyme exhibited a negligible maximum velocity (Vmax = 0.002 µM min^−1^), confirming its operational inability to function outside acidic environments. This stands in sharp contrast to its performance determined under standard assay conditions at pH 4, where the fungal laccase exhibits optimal activity with a Vmax of 1.6 µM min^−1^. Therefore, the Vmax of the natural laccase is nearly completely diminished at pH 6. This observation aligns with established literature for natural laccases; like all enzymes, their structural integrity is highly sensitive to storage conditions and transport, with activity diminishing drastically in neutral (over 80% loss activity) or alkaline media due to the destabilization of their active copper centers [34,35,36].

In contrast, the Mn-based nanozymes maintained high catalytic turnover, validating the protective role of the siloxane matrix against metal leaching and deactivation.

A comparative analysis of the calculated kinetic parameters (detailed in Table 4) between the 1MeIm-free and imidazole-functionalized systems reveals a distinct structure-function trade-off regarding substrate affinity (Km) and catalytic throughput (Vmax). The Mn-APTES-6 control displayed the highest substrate affinity (Km = 13.02 µM), likely driven by unhindered electrostatic interactions between the surface amine groups and the anionic substrate. Comparatively, the commercial fungal laccase exhibited a Km of 41.73 µM, a value consistent with the broad affinity range (22–2262 µM) reported for various T. versicolor isoenzymes [34]. However, the coordination of 1MeIm in the optimized Mn-APTES/1MeIm-6 nanozyme induced a fundamental kinetic shift that aligns with industrial remediation requirements. Although the affinity decreased (Km = 94.1 µM), this shift cannot be solely attributed to the steric hindrance introduced by the 1MeIm ligand. The coordination of the imidazole moiety fundamentally alters the electronic density of the manganese center, mimicking the natural histidine–metal interaction. This electronic modulation likely changes the binding mode and the free energy of substrate association, slightly reducing the affinity but significantly accelerating the rate-limiting electron transfer step, as evidenced by the surge in maximum reaction velocity (Vmax) to 4.331 µM min^−1^. This represents a nearly four-fold enhancement in turnover velocity compared to the control. Notably, while the natural enzyme lost >99% of its functionality at neutral pH, the nanozyme maintained high oxidative throughput, effectively overcoming the physiological limitations that restrict biocatalysts in wastewater environments. Crucially, this alteration highlights a functional specialization, while natural laccases are evolved for scavenging trace substrates (low Km), the engineered nanozyme prioritizes high-throughput turnover (high Vmax), a preferable trait for treating effluent streams with elevated pollutant loads. By maintaining unsaturated catalytic rates at higher concentrations, the nanozyme is effectively tuned for ‘bulk’ degradation, avoiding the rapid saturation inhibition that typically restricts natural biocatalysts in high-strength wastewater matrices.

### 2.4. Comparative Degradation of Oxytetracycline

**Figure 4:**
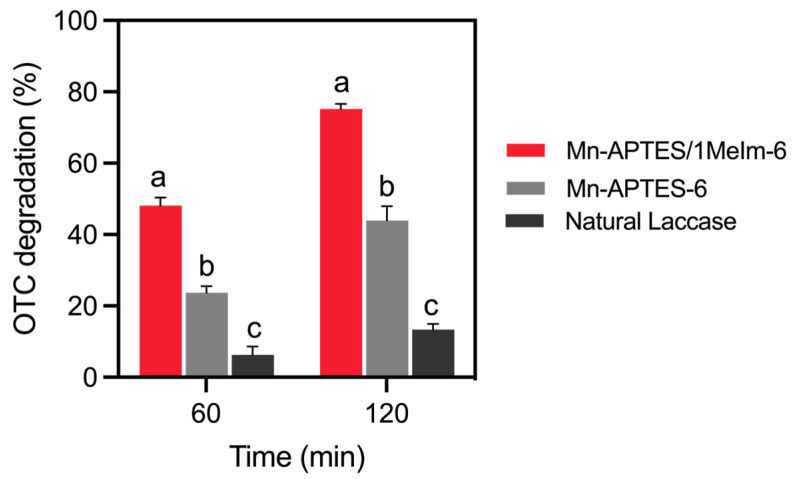
Comparative degradation of oxytetracycline (OTC) by Mn-APTES-6 and Mn-APTES/1MeIm-6 nanozymes versus commercial laccase. The degradation assays were conducted with an initial OTC concentration of 10 mg L^−1^, catalyst dose (1 mg ml^−1^), pH 6 under 200 rpm agitation at room temperature for 2 h. Different letters indicate statistically significant differences (*p* < 0.05) according to Tukey’s HSD post hoc test.

To validate the practical utility of the synthesized materials for environmental remediation, the catalytic efficacy of the optimized Mn-APTES/1MeIm-6 nanozyme was assessed through the degradation of oxytetracycline (OTC). Selected due to its extensive use in aquaculture and livestock breeding, where approximately 30–90% of the administered antibiotics are excreted as parent compounds capable of disrupting microbial ecosystems and entering the food chain, the removal of this persistent pollutant was monitored as illustrated in Figure 4. The comparative kinetic analysis demonstrated that the imidazole-functionalized hybrid significantly outperformed both the ligand-free Mn-APTES-6 assembly and the natural commercial laccase across all monitored time intervals. Within the first 60 min of the reaction, the Mn-APTES/1MeIm-6 system achieved approximately 48% OTC removal, effectively doubling the efficiency of the Mn-APTES-6 control (~23%) and exhibiting a nearly seven-fold enhancement over the native enzyme, which degraded less than 7% of the substrate. This superior activity persisted over the full 120 min duration, reaching a 75% total degradation for the complete hybrid system.

To distinguish catalytic oxidation from physical adsorption and auto-degradation, control assays were conducted under an inert nitrogen atmosphere and in the absence of the nanozyme. Control experiments containing only OTC in buffer under identical experimental conditions (room temperature, ambient light, 2 h) exhibited less than 1% degradation, confirming that auto-degradation via hydrolysis or photolysis was negligible. Furthermore, the nitrogen-purged tests revealed that physical adsorption accounted for only 6.3% of the OTC removal, confirming that the observed 75% reduction is primarily driven by the laccase-like oxidative degradation facilitated by the manganese active sites. In contrast, the Mn-APTES-6 precursor and the natural laccase reached early degradation plateaus of only 44% and 13%, respectively. The statistically significant performance gap (*p* < 0.05) observed between the imidazole-modified and unmodified nanozymes confirms that the coordination of 1MeIm is critical for modulating the manganese center’s redox potential and maximizing oxidative turnover. While strictly quantitative comparisons show that advanced systems like b-CoNi-MOF or NIR-assisted Cu-Mn nanozymes achieve marginally higher degradation rates (~80–90%) [18,37,38,39], these often necessitate external energy inputs (photothermal heating, UV light) or complex, energy-intensive synthesis protocols. In contrast, our Mn-APTES/1MeIm system achieves comparable remediation efficiency (75%) driven solely by the intrinsic redox properties of the architecturally stabilized manganese, operating effectively at room temperature without auxiliary photo-activation. This distinct ‘passive’ efficiency highlights the practical sustainability of the proposed nanozyme for real-world wastewater treatment, offering a robust alternative where energy costs and operational simplicity are critical constraints. Crucially, the structural robustness of the catalyst was corroborated via inductively coupled plasma–optical emission spectrometry (ICP-OES). Analysis of the supernatant following centrifugation and degradation assays revealed no detectable manganese leaching, confirming that the metal centers are strongly stabilized within the nanozyme architecture. This rigid encapsulation is further favored by the operational pH, which thermodynamically minimizes the propensity for manganese solubilization. Furthermore, the substantial underperformance of the natural laccase underscores the enhanced stability and kinetic proficiency of the synthetic mimetic, identifying it as a more robust candidate for wastewater treatment applications where biological enzymes typically falter.

While the OTC removal efficiency highlights the robust catalytic activity of the Mn-APTES/1MeIm-6 nanozyme, it is important to note that the current study focused on the primary degradation of the parent compound. Future investigations utilizing advanced LC-MS/MS techniques alongside biological inhibition assays are necessary to identify the intermediate transformation products and ensure that no secondary toxicity is generated during the remediation process.

### 2.5. Characterization of Mn-APTES/1MeIm-6 Nanozyme

**Figure 5:**
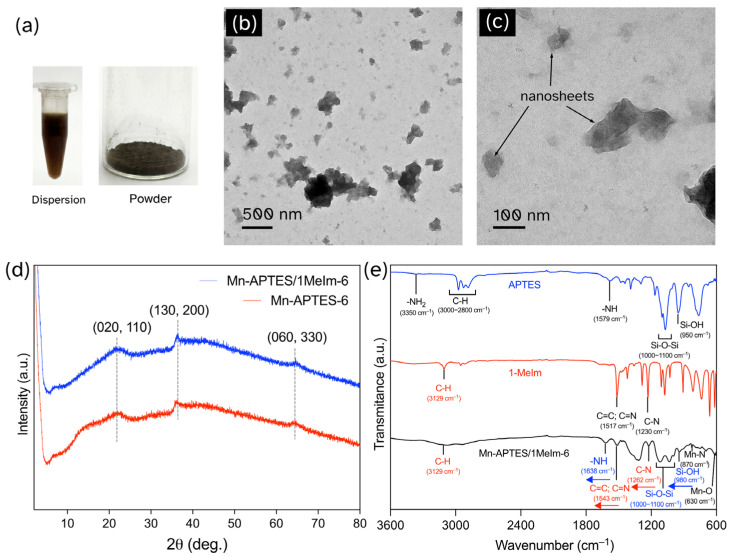
Characterization of Mn-APTES/1MeIm-6 nanozymes. (**a**) Photographs of the nanozymes in aqueous dispersion and powder form. (**b**,**c**) Transmission electron microscopy (TEM) images. (**d**) X-ray diffraction (XRD) pattern. (**e**) Fourier transform infrared (FTIR) spectra (blue and red arrows indicate shifts in functional groups in Mn-APTES/1MeIm-6 compared with pure APTES and 1-MeIm, respectively).

The physicochemical characteristics of the optimized Mn-APTES/1MeIm-6 nanozyme were established through various techniques, as summarized in Figure 5. Macroscopically (Figure 5a), the material appears as a homogeneous dark brown dispersion exhibiting significant colloidal stability. This is evidenced by a positive zeta potential of 27.7 ± 0.75 mV. This positive charge primarily originates from the protonation of the terminal primary amine groups (−NH_3_^+^) of the APTES molecules on the nanosheet surfaces at the experimental pH, providing strong electrostatic repulsion that prevents aggregation [20]. Transmission Electron Microscopy (TEM) images (Figure 5b,c) reveal that while the nanozyme presents as irregular aggregates, high-resolution imaging resolves these structures into discrete, electron-transparent nanosheets. This transparency confirms the successful formation of an ultrathin, layered two-dimensional framework distinct from bulk particulate matter. This morphology is indicative of a delaminated architecture, similar to that predominantly reported for organophyllosilicates or aminoclays, and contrasts with the characteristic shapes often observed in imidazole-based nanomaterials, such as nanorods [23], open vesicles [40], or yarn-ball-like structures [41].

The crystallographic information of the synthesized Mn-APTES-6 and Mn-APTES/1MeIm-6 was evaluated via X-ray diffraction (XRD) (Figure 5d). The diffraction patterns for both materials are characteristic of organophyllosilicates, evidencing a successful synthesis free from crystalline manganese oxide impurities. The XRD pattern is characteristic of a delaminated aminoclay-type structure, where the APTES-derived siloxane networks form the tetrahedral sheets, and the manganese centers occupy the octahedral coordination positions. This structural analogy solidifies the presence of a 2D organophyllosilicate framework. The experimental profiles were benchmarked against established literature values for manganese aminoclays, which typically display a basal spacing at d001 = 1.40 nm (2θ = 6.3°) and broad in-plane reflections at d020,110 = 0.39 nm (2θ = 22.9°) and d130,200 = 0.26 nm (2θ = 34.8°) [26].

Consistent with these reported values, the Mn-APTES-6 diffractogram exhibits the characteristic in-plane reflections centered near 23° and 35°, confirming the formation of the octahedral metal-oxide sheet. Notably, the crystallographic profile reveals a highly advantageous structural feature in the basal region. Unlike the literature benchmark, which typically exhibits a distinct reflection at 6.3° corresponding to a regular stacking order [37], the Mn-APTES-6 sample displays intense scattering at low angles (2θ < 4°) with the complete absence of the (001) diffraction peak. Far from being a structural defect, this indicates that the synthesized aminoclay has undergone complete delamination into randomly oriented, individual nanosheets. This high degree of exfoliation is critical for catalytic performance, as it maximizes the effective specific surface area and ensures that the manganese active sites remain sterically accessible to bulky substrates like ABTS and OTC. This structural openness directly correlates with the exceptional kinetic turnover (Vmax) observed, confirming that the optimized synthesis effectively prevents the stacking interactions that typically bury active sites in conventional layered materials. Consistent with this amorphous, delaminated state, no diffraction peaks associated with crystalline imidazole were observed in the Mn-APTES/1MeIm-6 pattern. This absence is attributed to the successful molecular dispersion of the ligand within the interlayer galleries, preventing its crystallization [41,42]. However, the reflection near 36° becomes slightly more defined in the Mn-APTES/1MeIm-6 spectrum, implying that the 1MeIm ligand coordinates effectively with the manganese centers, locally stabilizing the inorganic framework without disrupting the desirable delaminated morphology.

Fourier Transform Infrared Spectroscopy (FTIR) provided molecular-level evidence of the successful integration of the mixed-ligand architecture and the specific coordination environment of the manganese (Figure 5e). Comparison with the pure APTES precursor confirmed the effective polymerization of the silane, evidenced by the retention of the intense asymmetric stretching of Si–O–Si bonds between 1000 and 1100 cm^−1^ and the Si–OH vibration at 980 cm^−1^ in the final nanocomposite [20]. The persistence of this cross-linked siloxane network within the Mn-APTES/1MeIm-6 nanozyme validates the formation of an inorganic matrix that structurally anchors the metal core, thereby providing the necessary steric entrapment to mitigate manganese leaching risks at neutral pH.

**Scheme 1:**
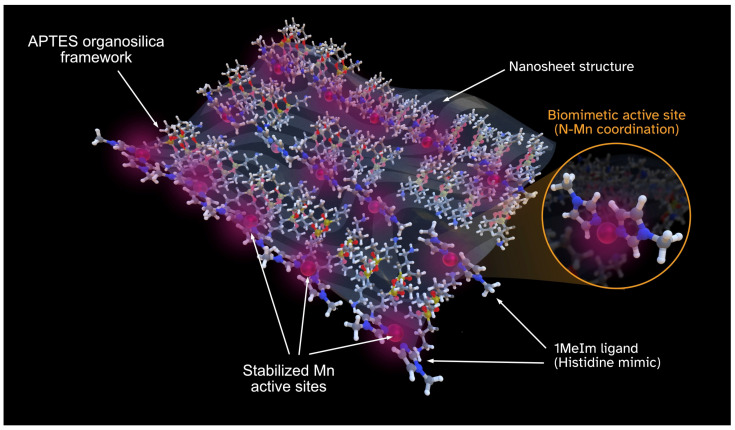
Schematic representation of the proposed structure of the Mn-APTES/1-MeIm nanozyme based on physicochemical characterization.

Alongside, the successful coordination of the 1MeIm ligand was substantiated by distinct shifts in the heteroaromatic ring vibrations relative to the free ligand. While pure 1MeIm exhibited characteristic C=C/C=N and C–N stretching modes at 1517 cm^−1^ [23] and 1230 cm^−1^ [42], respectively, these diagnostic bands underwent a significant blue shift to 1543 cm^−1^ and 1262 cm^−1^ in the Mn-APTES/1MeIm-6 nanozyme. This vibrational energy increase (Δν ≈ 26–32 cm^−1^) indicates a stiffening of the ring bonds resulting from the donation of nitrogen lone pair electrons to the manganese, effectively mimicking the metal–histidine interactions found in the active sites of natural metalloenzymes. Furthermore, the emergence of a discrete band at 630 cm^−1^, assigned to the Mn–O stretching vibration [21], provided direct confirmation of the manganese incorporation within the hybrid organosilica lattice. Collectively, these structural and spectroscopic findings support the proposed supramolecular architecture illustrated in Scheme 1, where the manganese active sites are chemically stabilized by ligand coordination and physically confined within the delaminated siloxane framework.

## 3. Materials and Methods

### 3.1. Chemicals and Reagents

All chemical reagents utilized were of analytical grade and employed without further purification. Manganese(II) nitrate tetrahydrate (Mn(NO_3_)_2_·4H_2_O) was purchased from Merck KGaA (Darmstadt, Germany). The organic ligands, 1-methylimidazole (1MeIm) and (3-aminopropyl)triethoxysilane (APTES), were obtained from Sigma-Aldrich Co. LLC (St. Louis, MO, USA). For the assessment of laccase-like activity, the chromogenic substrate 2,2′-azino-bis(3-ethylbenzothiazoline-6-sulfonic acid) (ABTS) was acquired from Sigma-Aldrich Co. LLC (St. Louis, MO, USA). Commercial fungal laccase from *Trametes versicolor* (≥0.5 U mg^−1^) was also obtained from Sigma-Aldrich for comparative purposes. HPLC-grade water was utilized for the preparation of all aqueous solutions and reaction mixtures.

### 3.2. Synthesis of Mn-APTES/1MeIm Nanozymes

The synthesis of the manganese-based nanozymes was performed via a modified coprecipitation method optimized through a central composite design. Initially, 1.5 mmol of Mn(NO_3_)_2_·4H_2_O was dissolved in 15 mL of absolute ethanol within a 20 mL glass vial and maintained under magnetic agitation at 200 rpm. Simultaneously, a specific mixture of ligands was prepared by combining varying molar ratios of APTES (3–9 mmol) and 1MeIm (3–9 mmol). This ligand solution was subsequently added dropwise to the manganese precursor solution. The reaction mixture was stirred continuously for 20 h under controlled thermal conditions (30–50 °C) as dictated by the experimental design. Upon completion of the synthesis, the resulting suspension was transferred to 50 mL tubes and centrifuged at 4100 rpm for 10 min to recover the solid precipitate. The supernatant was discarded, and the solids were subjected to three washing cycles with absolute ethanol, employing vortex agitation followed by centrifugation (5 min per cycle) to remove unreacted precursors. Finally, the purified pellets were dried on glass disks at 60 °C overnight and ground into a fine powder using a Teflon spatula and an agate mortar prior to storage.

### 3.3. Physicochemical Characterization

The synthesized nanozymes were characterized using a suite of analytical techniques to elucidate their structural, morphological, and surface properties. X-ray diffraction (XRD) analysis was conducted using a Bruker D8 Advance diffractometer equipped with a LynxEye linear detector (Bruker, Karlsruhe, Germany), operating with Cu Kα radiation (40 kV/30 mA). Morphological features were examined via transmission electron microscopy (TEM) using a JEOL 1200 EX II microscope (JEOL Ltd., Tokyo, Japan). To assess colloidal stability in aqueous suspension, the hydrodynamic diameter and zeta potential were determined via dynamic light scattering (DLS) using a Zetasizer Nano ZS90 (Malvern Instruments, Worcestershire, UK). Furthermore, Fourier transform infrared spectroscopy (FTIR) was performed using an Agilent Cary 630 FTIR spectrophotometer (Agilent Technologies, Santa Clara, CA, USA) to identify functional groups and confirm ligand coordination.

### 3.4. Catalytic Activity and Kinetic Analysis

The laccase-like activity was quantified spectrophotometrically by monitoring the oxidation of ABTS at ambient temperature [11]. Reaction assays were prepared in phosphate buffer at pH 6, 7, and 8, maintaining a final substrate concentration of 100 µM and a catalyst concentration of 50 µg mL^−1^. The reaction kinetics were monitored by recording the absorbance at 420 nm for the oxidized ABTS product using a Spectroquant Prove 300 spectrophotometer (Merck KGaA, Darmstadt, Germany) at 5 s intervals over a 1.5 min period. Steady-state kinetic parameters (Vmax and Km) were determined by measuring the initial reaction velocities (V0) across a substrate concentration range of 10–250 µM and fitting the data to the Michaelis–Menten equation.

### 3.5. Degradation Assays of Oxytetracycline

The catalytic degradation efficiency was evaluated using the optimized Mn-based imidazole nanozyme against tetracycline antibiotics, specifically oxytetracycline (OTC). The reaction mixtures consisted of a 10 mg L^−1^ antibiotic solution prepared in distilled water, with the pH adjusted to the optimum activity level previously determined, and nanozyme concentrations ranging from 0.1 to 0.5 g L^−1^. Samples were collected at 30 min intervals over a duration of 6 h and were subsequently filtered through a 0.20 µm membrane prior to analysis. The residual concentration of tetracyclines was quantified using an Agilent 1260 Infinity II high-performance liquid chromatography (HPLC) system equipped with a UV detector set at 280 nm [5]. Separation was achieved on an Agilent C18 column (150 mm × 4.6 mm, 5 µm, Agilent, Santa Clara, CA, USA) using a mobile phase of 0.02 M phosphoric acid and acetonitrile (80:20, *v*/*v*) delivered at a flow rate of 1.0 mL min^−1^. To differentiate between catalytic degradation and physical adsorption, parallel control experiments were conducted using tetracycline solutions purged with nitrogen gas for 20 min at a flow rate of 25 mL s^−1^. Additionally, to assess structural stability during the process, potential metal leaching was monitored via inductively coupled plasma–optical emission spectrometry (ICP-OES) using an Agilent 5110 instrument (Santa Clara, CA, USA).

## 4. Conclusions

This study demonstrates the rational design of a Mn-APTES/1MeIm nanozyme that successfully stabilizes manganese active sites at neutral pH, overcoming the rapid inactivation typical of natural laccases. Response Surface Methodology revealed distinct synthesis mechanisms: catalytic activity at pH 6 is thermally driven by morphology, whereas activity at pH 7 is stoichiometrically controlled by ligand shielding. Functionally, the optimized nanozyme exhibited superior kinetics, with a maximum reaction velocity (Vmax) over 2000-fold higher than the commercial fungal laccase. In practical application, the Mn-APTES/1MeIm-6 nanozyme achieved 75% degradation of oxytetracycline in 120 min without external energy input, significantly outperforming the natural enzyme (<13%). These results confirm the nanozyme as a robust, cost-effective alternative for the remediation of recalcitrant antibiotics in wastewater. Future investigations will focus on assessing the operational stability of the catalyst over multiple degradation cycles to establish its reusability in complex wastewater matrices and evaluating the ecotoxicity of the degradation intermediates to fully establish the operational lifecycle of the nanozyme.

## Figures and Tables

**Table 1:** Response Surface Methodology (RSM) through a Central Composite Design (CCD), including three central points, for the maximization of laccase-like activity in the Mn–1MeIm/APTES nanozyme. Experimental and predicted values for laccase-like activity using ABTS as a substrate at pH 6 and pH 7.

Synthesis No.	Coded Levels			Real Levels			Responses			
	*X*_1_	*X*_2_	*X*_3_	*X*_1_	*X*_2_	*X*_3_	*Y*_1_		*Y*_2_	
	APTES (mmol)	1MeIm (mmol)	Temp (°C)	APTES (mmol)	1MeIm (mmol)	Temp (°C)	Laccase-like Activity (U mg^−1^) at pH 6		Laccase-like Activity (U mg^−1^) at pH 7	
							Exp. Value	Pred. Value	Exp. Value	Pred. Value
1	0	1	0	6	9	40	0.18	0.42	0.95	1.08
2	−1	0	0	3	6	40	0.02	0.18	0.85	0.60
3	+1	+1	−1	9	9	30	1.73	1.68	0.92	0.71
4	+1	−1	−1	9	3	30	2.75	2.50	0.65	0.60
5	0	0	0	6	6	40	0.62	0.46	1.30	1.18
6	0	−1	0	6	3	40	1.02	0.88	1.60	1.58
7	0	0	−1	6	6	30	1.25	1.90	0.72	0.95
8	+1	+1	+1	9	9	50	0.65	0.63	0.82	0.82
9	0	+1	0	3	9	30	1.98	1.57	0.06	0.06
10	0	0	+1	3	3	50	0.65	0.67	0.95	1.14
11	+1	0	+1	9	3	50	0.55	0.93	0.92	0.83
12	−1	−1	−1	3	3	30	2.20	2.20	1.08	1.05
13	0	0	0	6	6	40	0.42	0.46	1.20	1.18
14	+1	0	0	9	6	40	0.42	0.37	0.40	0.76
15	−1	+1	+1	3	9	50	0.32	0.56	0.02	0.02
16	0	0	0	6	6	40	1.30	0.46	0.58	1.18
17	0	0	+1	6	6	50	1.30	0.68	1.15	1.04

One unit (U) of laccase-like activity is defined as the amount of nanozyme required to oxidize 1 µmol of ABTS per minute under the specified assay conditions.

**Table 2:** Summary of model fit and analysis of variance (ANOVA) for nanozyme activity.

Statistical Parameter	Model for Laccase-like Activity	
	pH 6	pH 7
Coefficient of determination (R^2^)	0.848	0.864
Adjusted R^2^	0.652	0.690
Root means square error (RMSE)	0.459	0.238
Model F-ratio	4.330	4.960
Model *p*-value	* 0.033	* 0.023

* Significant at the 95% confidence level (*p* < 0.05).

**Table 3:** Optimized synthesis parameters and comparison of predicted vs. validated laccase-like activity.

Nanozyme	Optimal Synthesis Conditions			Predicted Activity (U mg^−1^)	Validated Activity (U mg^−1^)
	APTES (mM)	1MeIm (mM)	Temp. (°C)		
Mn-APTES/1MeIm-6	7.24	3	30	2.55	2.23
Mn-APTES/1MeIm-7	5.45	3	42	1.61	1.45

**Table 4:** Kinetic parameters for substrate oxidation at neutral pH.

Catalyst	Vmax (µM min^−1^)	Km (µM)
Natural laccase	0.002	41.73
Mn-APTES-6	1.155	13.02
Mn-APTES/1MeIm-6	4.331	94.1
Mn-APTES-7	0.890	48.21
Mn-APTES/1MeIm-7	1.710	82.86

## Data Availability

The original contributions presented in this study are included in the article. Further inquiries can be directed to the corresponding author.

## Abbreviations

The following abbreviations are used in this manuscript:

Abbreviation	Full Name
RSM	Response Surface Methodology
CCD	Central Composite Design
APTES	(3-Aminopropyl)triethoxysilane
1MeIm	1-Methylimidazole
OTC	Oxytetracycline
ABTS	2,2′-Azino-bis(3-ethylbenzothiazoline-6-sulfonic acid)
WWTP	Wastewater Treatment Plant
XRD	X-Ray Diffraction
TEM	Transmission Electron Microscopy
FTIR	Fourier Transform Infrared Spectroscopy
DLS	Dynamic Light Scattering
ICP-OES	Inductively Coupled Plasma–Optical Emission Spectrometry
HPLC	High-Performance Liquid Chromatography
MOF	Metal–Organic Framework
